# Assessing the carbon footprint of the initial 24 h post-severe trauma admission in a French ICU: a pilot study

**DOI:** 10.1186/s13613-025-01536-x

**Published:** 2025-08-11

**Authors:** Candice Marion, Matthieu Bernat, Emmanuelle Hammad, Jean-Paul Calvet, Manon Roche, Ludivine Marecal, Laurent Zieleskiewicz, Marc Leone

**Affiliations:** 1 Department of Anaesthesia and Intensive Care Medicine, Hôpital Nord, Assistance Publique des Hôpitaux de Marseille, Aix Marseille University, Marseille, France; 2 École nationale supérieure d’Arts et Métiers, Aix-en-Provence, France; 3 Pharmacy Department, Service Central des Opérations Pharmaceutiques, Hôpital Nord, Assistance Publique des Hôpitaux de Marseille, Aix Marseille University, UMR CNRS 7273, Marseille, France

**Keywords:** Climate change, Global warming, Greenhouse gas, Carbon footprint, Environment, Trauma, Intensive care

## Abstract

**Background:**

As healthcare emerges as the world’s fifth-largest carbon emitter, intensive care units (ICUs) represent environmental challenges due to their high resource consumption and energy demands. Reducing greenhouse gas (GHG) emissions is necessary to limit global warming. This study aimed to quantify the carbon footprint of ICU care during the first 24 h of admission for trauma patients. By establishing a baseline “carbon cost” for ICU trauma care, we seek to provide a framework for future studies assessing sustainable care strategies.

**Methods:**

We conducted a prospective observational pilot study in a French trauma ICU, categorizing patients into three standardized care pathways. The GHG emissions have been quantified using a hybrid life cycle assessment approach across various scope categories. Statistical analyses included correlation testing between the different groups and severity scores.

**Results:**

Total carbon footprints ranged from 86 to 248 kg of CO_2_e per patient over the first 24 h. Medications, medical devices, and transportation were the primary contributors, while energy and waste represented a smaller portion of the emissions. There was a significant positive correlation between emissions and severity scores.

**Conclusion:**

The carbon footprint of ICU care of a trauma patient during the first 24 h is significant, and it is necessary to conduct assessments in each ICU to identify levers for environmental improvement. The carbon cost should be integrated into the standardization of care and research protocols to enable more sustainable care practices.

**Supplementary Information:**

The online version contains supplementary material available at 10.1186/s13613-025-01536-x.

## Introduction

Climate change poses a paramount threat to our planet, affecting ecosystems, economies, and human health. The surge in greenhouse gas emissions (GHG), largely driven by human activities, has led to unprecedented climatic shifts [1, 2]. Healthcare accounts for 4.4% of global net emissions annually, equivalent to 2 gigatons of carbon dioxide (CO_2_) [3]. At this rate, the healthcare sector would rank as the fifth-largest emitter in the world [4]. Reducing GHG emissions is essential to limit global warming.

Within healthcare, the intensive care unit (ICU) significantly contributes to global warming due to continuous activity, resource consumption, and energy demand [3]. Several studies have examined anesthesia-related GHG emissions, notably due to halogenated gases [5, 6]. However, limited research has focused on ICU management, with a notable gap in understanding the carbon footprint of ICU care in trauma settings [7].

Our primary objective was to quantify the average CO_2_ emissions during the first 24 h of ICU admission for severe trauma across three standardized care pathways with increasing severities. Our secondary objectives were to examine the correlation between severity scores and CO_2_ emissions, to calculate CO_2_ emissions by category of emission in each group, to compare CO_2_ emissions across groups, and to assess specific CO_2_ emissions associated with medications and energy across groups. In addition, as healthcare sustainability encompasses GHG emissions, we compared the average plastic and rubber weight used in each group.

## Materials and methods

### Population and case location

We conducted a prospective observational pilot study in the trauma ICU of a French university hospital. The facility encompasses 15 ICU beds along with a level-1 trauma center. Our study focused on adult patients admitted for severe trauma from May 1, 2023, to February 29, 2024. Severe trauma was defined on the criteria detailed in the Field Triage Decision Scheme from the American College of Surgeons Committee on Trauma (ACS-COT) [8].

Three standardized scenarios were developed based on clinical experience within our ICU. These scenarios reflect a presupposed increase in the use of medical devices, medications, and resources required for patient care:

- *Group mild*: Patient admitted for severe trauma with hemodynamic, respiratory, and neurological stability at the ICU admission. Subsequently, they were categorized as requiring “low” resources: minimal equipment, few additional examinations, no specific resuscitation devices, and oral medication administration.
- *Group intermediate*: Patient admitted for severe trauma with traumatic brain injury as a single injury and requiring vascular lines, monitoring devices, invasive mechanical ventilation, and intravenous medication administration.
- *Group severe*: Patient admitted for severe trauma with acute respiratory failure and severe bleeding requiring vascular lines, invasive mechanical ventilation, additional examinations (multiple computed tomography (CT) scan or blood samples), extensive use of medical devices (MD), and intravenous medications including massive transfusion.

We enrolled patients upon their admission in the trauma bay, categorizing them into one of the three pathways at the end of the 24-hour period. A convenience sample of nine trauma patients was predetermined, with three patients allocated to each group. No sample size calculation was performed, as no a priori knowledge of any environmental footprint data was available. However, it was considered that nine patients would be sufficient to provide a reasonable data range. Patients were chosen sequentially, considering the expected type of trauma and the availability of investigators. For each patient, comprehensive data on trauma mechanisms and scoring systems including simplified acute physiology score (SAPS) II, sequential organ failure assessment (SOFA), and injury severity score (ISS) were meticulously collected.

### Ethical considerations

According to the French law, our study has been classified as research not involving human subjects, and was approved by our local ethic committee (n°CSE24-4) [9]. Patients were given written information about our research when they were discharged from the ICU and were able to opt out of having their data used.

### Study scope

We conducted a carbon footprint assessment in accordance with the International Organization for Standardization (ISO 14040) methodology [10]. We defined a frame corresponding to the patient management in our ICU. Next, we defined emissions categories, including medical devices, medications, staff and patient nutrition, medical or digital devices, ICU infrastructures, medical imaging and laboratory, energy, staff and visitor transportation, and waste.

**Fig. 1:**
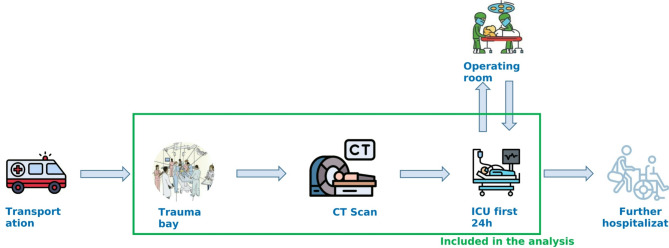
Scope of the carbon footprint analysis

We excluded the patient transportation, the external services (laundry, security) and the patient management in the operating room as they constitute separate hospital activities (Fig. 1).

### Calculation methods

#### GHG emissions

We calculated GHG emissions in carbon dioxide equivalents (CO_2_e), a standardized metric that enables the comparison of different GHGs to CO_2_ based on their 100-year Global Warming Potential (GWP100). The carbon footprint of the studied system was determined using the following formula:

$$\eqalign{& {\rm{Greenhouse}}\>{\rm{Gas}}\>{\rm{Emissions}} \cr & {\rm{ = Activity}}\>{\rm{Data \times Emission}}\>{\rm{Factor}}\left( {{\rm{EF}}} \right) \cr} $$

Activity data represent the quantity of a given activity (e.g., energy usage, material consumption), while the emission factor indicates the carbon intensity associated with that activity [11]. We quantified the activities of the various scopes within our ICU by gathering field or microeconomic data (bottom-up approach) [12].

#### Emission factors

A comprehensive database for all medical devices and activities does not currently exist. To assess the environmental impact accurately, we relied on life cycle assessments (LCA), a methodology that evaluates environmental effects across a product’s entire life cycle—from raw material extraction to disposal (“cradle to grave”) [13].

In our study, we employed a process-based LCA approach, which relies on directly obtained, specific data for environmental impact analysis. This variety of LCA was invariably favored on account of its precision. Exceptionally, when process-based LCA data was unavailable, we used economic input-output LCAs (EIO-LCA), which estimate environmental impacts based on the economic cost of activities [14, 15]. This approach was employed only for three specific medications (see S1 file).

The primary source of EF was the ADEME Base Empreinte database [16]. We also incorporated data from prior studies that conducted LCAs for specific products within the scope of our study. Detailed emission factors and sources are listed in the S1 File, Appendices A1 to A4.

#### Uncertainty analysis

Medications and MDs are among the highest contributors to carbon emissions in the healthcare sector [17]. To address uncertainty, as recommended by ISO 14,040, we assigned each EF in the MD and medication inventory to a level within a 3-tier pedigree matrix, based on the robustness and origin of the underlying data [10, 18]:

Level 1– Full process-based LCA (± 10% uncertainty): Transparent, published, peer-reviewed LCA studies conforming to ISO 14,040 standards;

Level 2– Semi-structured tool-based estimation (± 30% uncertainty): Emission factors generated using tools such as Carebone AP-HP, which provide partial LCAs (e.g., differentiating API, packaging, transport) but omit certain stages like API manufacturing;

Level 3– Economic input-output (EIO) based estimation (± 100% uncertainty): Monetary proxies derived from cost-based emissions, lacking process-specific details, and subject to wide variation.

These uncertainty levels were applied to each MD and medication in our inventory and computed in a sensitivity analysis providing both worst-case and best-case scenarios.

### Data collection for different study post

#### Medical devices

We used single-use medical devices, discarded after use. Quantities were tallied from waste bins and cross-checked with prescription records. To estimate the EF, the following method was used:

$${\bf{MD}}\,{\bf{EF}} = {\bf{Production}}\,{\bf{EF}} + {\bf{Transport}}\,{\bf{EF}}$$

$${\rm{Production}}\,{\rm{EF = mass}}\left({{\rm{kg}}} \right)*\:{\rm{material}}\,{\rm{EF}}$$

$$\eqalign{& {\rm{Transport}}{\mkern 1mu} {\rm{EF}} \cr & {\rm{ = mass}}{\mkern 1mu} \left( {{\rm{kg}}} \right){\rm{*}}\,{\rm{distance}}\left( {{\rm{km}}} \right){\rm{*}}\,{\rm{transport}}{\mkern 1mu} {\rm{EF}} \cr} $$

Material composition and production locations were sourced from product datasheets or packaging. We measured the weights of both the device materials and their packaging using a scale with 0.01 g accuracy. Material EFs were extracted from the ADEME database [16]. For transportation, distances were calculated via SeaRates, assuming truck transport within the EU and container shipping for longer routes [19]. Given the lack of specific data, sterilization of DM was not included in the analysis.

#### Medications

We relied on computerized prescriptions. The EF was obtained from process-based LCAs studies or databases such as Carebone AP-HP Primum Non Nocere and ECOVAMED when available [20]. For midazolam, tranexamic acid and insulin, no process-based LCAs were available. Hence, EF was estimated using the EIO-LCA for pharmaceutical products provided by The Shift Project, set at 300 kg CO_2_e per €1,000 (tax free) [17]. Medication costs were extracted from the ICU ordering software.

#### Nutrition

In our center, patients do not consume food during the first 24 h, either due to fasting or sedation with intubation. For healthcare providers, we estimated that each patient had two dedicated individuals, resulting in four meals over 24 h. We used the EF from ADEME for one average meal [16].

#### Medical imaging and laboratory

For imaging, the procedure is standardized for all incoming patients to the ICU including a Focused Assessment with Sonography in Trauma (FAST), chest X-rays, and a body CT scan. The number of biological assessments was obtained from the laboratory software. EF used were derived from McAlister’s process-based LCA studies [21, 22].

#### ICU infrastructure

Data were obtained from the layout plan of the ICU. The unit comprises 15 rooms covering a total area of 300 m^2^, estimated at 20 m^2^ per patient including common space. Emissions were calculated using the methodology from the Shift Project, which provides an EF per square meter of construction adjusted over a 30-year amortization period [12].

#### Medical equipment or digital devices

We inventoried medical equipment in the ICU. For digital devices such as computers, EFs were sourced from ADEME [16]. For specialized medical equipment such as ventilators and hospital beds, emissions were calculated using an economic allocation approach linked to the devices’ expected lifespan.

#### Energy

Electricity consumption was recorded in kilowatt-hours (kWh) based on device usage over 24 h. Gas consumption was estimated from the building’s annual usage, as no dedicated meter exists for the ICU. Oxygen consumption was calculated using the fraction of inspired oxygen (FiO_2_) and ventilator flow rates. Emission factors for medical oxygen were taken from the Climat Mundi process-based LCA database (AP-HP) [20].

#### Staff and visitor transportation

Transportation-related emissions were based on an assumption of two dedicated staff members and one visitor per patient. Given the hospital’s suburban location and limited public transport options, most transports were made by private car. A round-trip distance of 20 km per journey was assumed based on an institutional survey, with emission factors sourced from ADEME [16].

#### Waste

Waste was categorized into general waste and infectious healthcare waste. Bins were weighed, and emission factors for waste treatment were obtained from the Shift Project [12]. The weight analysis of plastic and rubber included materials such as polypropylene, polyethylene, polyvinyl chloride, polyester, nitrile rubber, and butyl rubber.

### Data analysis

No sample size was pre-defined due to the explorative feature of our study. Calculations were performed using Python 3.12 for data processing. Normally distributed data were represented with means, standard deviations, and 95% confidence intervals (CI), determined by the Shapiro-Wilk test for normality, while skewed data were summarized using medians and interquartile ranges (IQR). Homogeneity of variances was tested using Levene’s test. Continuous variable group comparisons were performed using either ANOVA followed by post-hoc Tukey tests, or the Kruskal-Wallis test followed by post hoc Dunn tests, as appropriate. Correlation between variables was assessed using Pearson’s correlation coefficient. All p-values were two-sided, with statistical significance defined as *p* < 0.05.

## Results

During the study period, three trauma patients were included in each group, for a total of nine patients. The demographic and clinical characteristics of the patients are detailed in Table 1. Group mild comprised younger patients with lower severity scores, while group severe included older patients with significantly higher injury and severity scores.

**Table 1:** Patient characteristics at ICU admission

	Group mild			Group intermediate			Group severe		
Patient	P1	P2	P3	P4	P5	P6	P7	P8	P9
Age (years)	33	47	46	28	78	33	78	66	48
Gender	M	F	F	M	M	M	M	F	M
Mechanism of trauma	Fall	Road traffic accident	Road traffic accident	Road traffic accident	Road traffic accident	Stab wound	Fall	Road traffic accident	Road traffic accident
Pre-hospital Grading ^a^	C	C	C	B	B	B	A	A	A
Intubation	No	No	No	Yes	Yes	Yes	Yes	Yes	Yes
SAPS II ^b^	8	19	15	30	46	17	76	74	74
SOFA score ^c^	0	0	0	6	6	4	12	13	10
ISS (AIS) ^d^	34	6	20	13	18	9	66	50	48

^a^ Pre-hospital grading locally classifies patients as grade A (unstable), grade B (stabilized) and grade C (stable) [8]

^b^ SAPS II (Simplified Severity Index Score) provides an estimate of the risk of death for patient in ICU [23]

^c^ The Sequential Organ Failure Assessment score allows for the assessment of severity and progression of organ failure in intensive care unit (ICU) patients [24]

^d^ The Injury Severity Score is specifically designed for the assessment of polytrauma patients. There is a strong correlation between this score and mortality, morbidity, and length of hospital stay [25]

### Total CO_2_e emissions and correlation with gravity scores

**Fig. 2:**
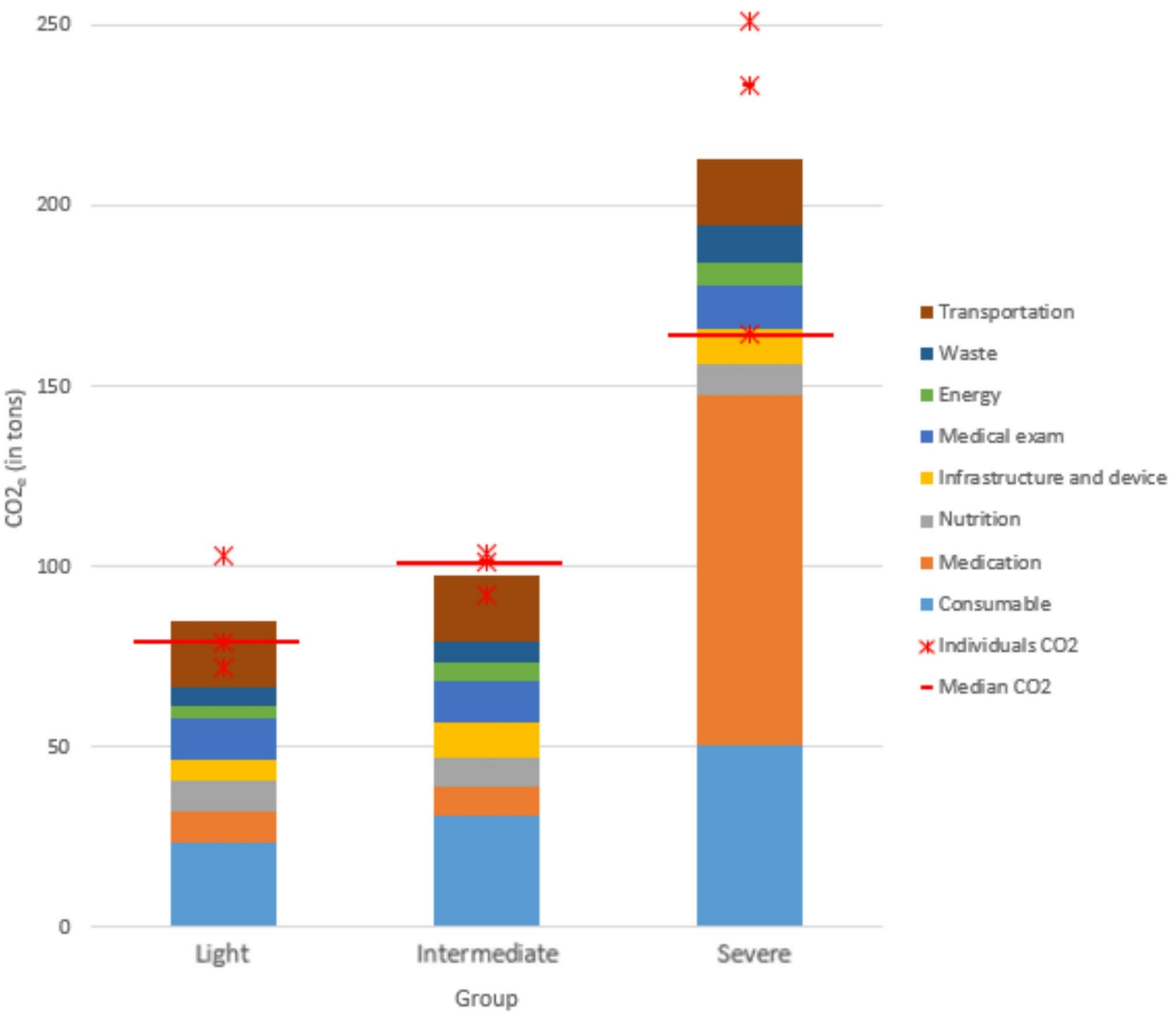
Distribution of total CO_2_e emissions in each group

During the initial 24 h after ICU admission, the carbon footprint was 85 ± 16 kg of CO_2_e, 98 ± 6 kg of CO_2_e, and 213 ± 46 kg of CO_2_e for group mild, intermediate, and severe, respectively (Fig. 2). Specifically, the group severe had substantially higher carbon footprint compared to the groups mild and intermediate. This difference between the three groups was primarily attributed to the higher use of medical devices and medications in the group severe than in the other groups.

Moreover, a significant strong positive correlation was observed between the severity scores and the CO_2_e emissions, with correlation coefficients as follows: *r* = 0.87, *p* < 0.01 for SAPS II; *r* = 0.86, *p* < 0.01 for SOFA; and *r* = 0.79, *p* < 0.05 for ISS (Fig. 2).

### CO_2_e emissions of different study categories

#### Global data

The total CO_2_e emissions per group and category are presented in Table 2. In average, MDs and medications were the highest contributors to the carbon footprint. In the group severe, medications accounted for 47% of the total emissions while the distribution of causes was more homogeneous in the groups mild and intermediate. The significant differences observed in emissions between the groups were primarily attributable to the increased use of MDs and medications in the group severe. Details are available in S1 File, Appendix A5.

**Table 2:** CO_2_e emissions for each defined category by group (in kg of CO_2_e). *Values are mean ± sd*

Category		Group mild	Group intermediate	Group severe
Entrance material	MD [Best case; worst case]*	24 [17; 31] **±** 11	31 [22; 40] ± 4	50 [35; 65] ± 10
	Medication [Best case; worst case]*	9 [6; 16] ± 3	8 [5; 15] ± 1	97 [79; 120] ± 35
	Nutrition	8 ± 0	8 ± 0	8 ± 0
Infrastructure and device	Building	2 ± 0	2 ± 0	2 ± 0
	Digital and medical equipment	4 ± 0	8 ± 0	8 ± 0
Medical exam	Imaging	11 ± 0	11 ± 0	11 ± 0
	Biological assays	0 ± 0	0 ± 0	1 ± 0.2
Energy	Electricity	2 ± 0	2 ± 0	3 ± 0
	Gas	1 ± 0	1 ± 0	1 ± 0
	Oxygen	0 ± 0	1 ± 0	3 ± 0
Waste	General	3 ± 0	4 ± 1	5 ± 3
	Infectious risk	2 ± 2	2 ± 1	5 ± 3
Transportation	Staff and visitor	18 ± 0	18 ± 0	18 ± 0
Total		85 ± 16	98 ± 6	213 ± 46

*Best and worst cases are calculated based on ISO 14,040 and GHG protocol pedigree matrix [10, 18]

#### Medical devices

The quantity of MDs showed a continuum of increase in the three groups (24 **±** 11 kg of CO_2_e (27% of total emissions for group mild, 31 ± 4 kg of CO_2_e (30%) for group intermediate, and 50 ± 10 kg of CO_2_e (20%) for group severe; *p* < 0.01). MDs were the primary source of GHG emissions in the mild and intermediate groups, and the second largest contributor in the severe group. Vascular access and perfusion elements accounted for the largest components. Details are available in S1 File, Appendix A5.

#### Medications

**Fig. 3:**
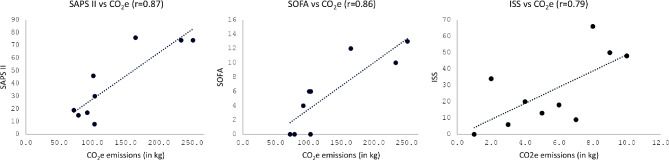
Correlation between total CO_2_e emissions and severity scores

Medications contributed to 9 ± 3 kg of CO_2_e (10%) in the group mild, 8 ± 1 kg of CO_2_e (8%) in the group intermediate, and 97 ± 35 (46%) in the group severe. The substantial increase in the group severe was due to the labile blood products, which represented 80% of medication-related emissions. The distribution of different medication group is available on Fig. 3. Details are available in S1 File, Appendix A5.

#### Energy

Energy consumption from gas was constant across the three groups at 1 ± 0 kg of CO_2_e, while electricity use increased from 2 ± 0 kg of CO_2_e in group mild to 3 ± 0 kg of CO_2_e in group severe. Energy constituted 4%, 5% and 3% of total emissions in the group mild, intermediate, and severe, representing a minor emission category. Details are available in S1 File, Appendix A5.

### Plastic and rubber weight

The average weight of plastic and rubber used per patient increased across groups, with group Mild using 6 ± 0 kg, group Intermediate using 8 ± 0 kg, and group Severe also using 12 ± 0 kg. Details are available in S1 File, Appendix A6.

## Discussion

**Fig. 4:**
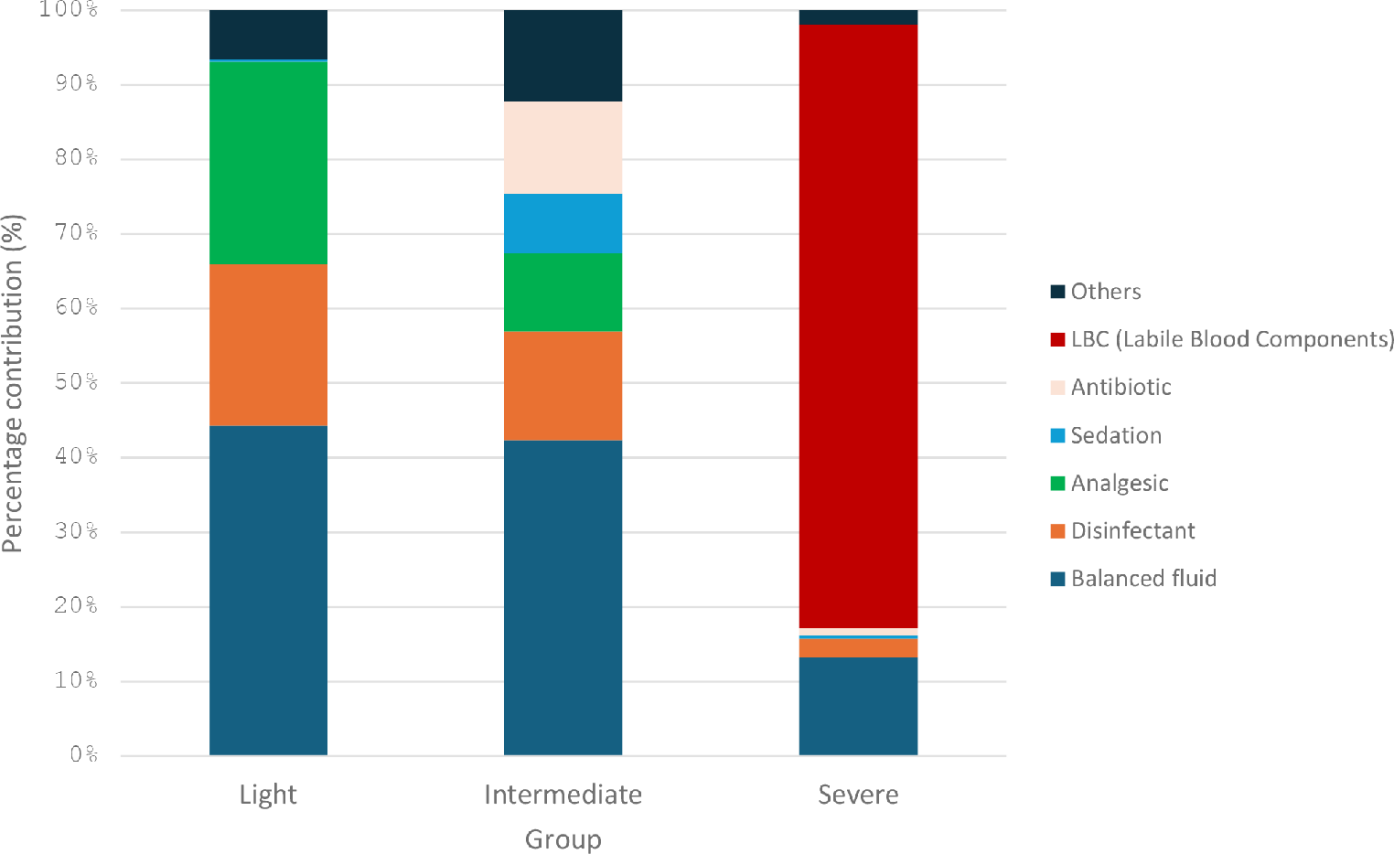
CO_2_e emission distribution of medications used in ICU across different groups

Our study quantified the carbon footprint associated with the first 24 h after ICU admission in trauma patients, stratified into three groups based on severity. To our knowledge, this represents one of the first and most comprehensive analysis of the carbon footprint in trauma ICU patients [7]. The average carbon footprint ranged from 86 to 248 kg of CO_2_​e, with a significant positive correlation between the severity scores and the CO_2_e emissions, equivalent to the emissions from driving a conventional car from 395 to 1,140 km. These findings align with previous research in ICU settings. For example, McGain et al. reported ICU carbon emissions of 88 to 178 kg CO_2_​e per patient per day in studies conducted in Australia and the USA. Similarly, Prasad et al. found that treating ICU patients in the USA was associated with an average carbon footprint of 138 kg of CO_2_​e per day per patient. Nevertheless, although our findings are within the same range as previous studies, methodological differences — particularly in the inclusion of single-use materials and differences in national energy mixes — may partly explain the variations observed in each emission category (Fig. 4).

Categorizing GHG emissions enables to identify the primary sources associated with the management of trauma patients. In both the mild and intermediate severity groups, MDs emerged as the dominant source of GHG emissions. All these MDs were single-use plastics, with no use of reusable materials. While transportation-related emissions for MDs were relatively minor, production processes accounted for a significant proportion of their carbon footprint. Moreover, in our analysis, approximately 5 to 12 kg of single-use plastics and rubber materials were used per patient during the first 24 h following trauma admission. Beyond their contribution to GHG emissions, plastic wastes pose additional health risks, including increased susceptibility to infectious diseases and heightened cardiovascular risk [26–28]. To mitigate these effects, several studies have recommended a transition to reusable MDs [29–31]. However, some reusable devices have been found to be less environmentally friendly than disposables, primarily due to the energy-intensive sterilization processes required for reuse in regions where energy production relies heavily on carbon-intensive sources [32, 33]. Nevertheless, in France, where nuclear energy predominates, reusable devices should be preferred to reduce both the carbon footprint and plastic consumption [29, 34].

In the public health sectors, medication purchases are a major emission source, ranging from 10% in the US to 29% in France [12, 35]. Because few process-based LCAs are available for medication, they are often excluded from the carbon footprint studies. Nevertheless, when including EIO-LCA, their contribution to emissions surpassed those of energy and MD [36]. In our study, we used an EF of 0.3 kg CO_2_e/eur from a French database [12, 14]. Medications accounted for approximately 10 kg of CO_2_e in the mild and intermediate severity groups and 132 kg of CO_2_e in the severe group. Based on a recent process-based LCA by Hibbs et al. this significant increase in the severe group was largely attributed to blood transfusions, which represented 80% of medication-related emissions [37]. To our knowledge, this is the first time that RBC transfusion has emerged as a major environmental impact. This is particularly significant, as 15% of severely injured trauma patients require blood transfusion, with 2–3% receiving massive transfusion defined as requirements for more than 10 units [38]. Interestingly, recent studies reported conflicting findings. Two randomized controlled trials showed that a liberal transfusion strategy in trauma patients does not yield better outcomes, as compared to a restrictive strategy [39, 40]. In contrast, Taccone et al. found that a restrictive strategy resulted on poorer outcomes, as compared to a liberal strategy [41]. Regarding these conflicting results, considering the carbon footprint may be included in the analysis.

Additionally, the high medication-related emissions in ICUs are largely driven by the extensive use of intravenous (IV) medications. IV drugs have a much higher carbon footprint than their oral counterparts due to the additional resources required for packaging, administration devices, sterilization, and storage. For instance, the carbon footprint of 1 g of oral paracetamol is 0.053 kg CO_2_​/unit, whereas the equivalent IV dose has an impact of 1.553 kg CO_2_e​/unit [42]. In many cases, the oral route is as effective as the IV route for administering certain medications. Prioritizing the oral route whenever feasible can further help to reduce GHG emissions while maintaining high standards of patient care. This could be achieved using tools such as a daily checklist to evaluate the necessity of IV medication, as proposed in our center [43].

In our analysis, the energy category encompassed electricity, gas, and oxygen consumption. Energy contributed only 3–5% of the total CO_2​_e emissions in our ICU, highlighting its relatively minor role. In contrast, studies from the USA and Australia reported average electricity consumptions of 44 and 124 kWh per patient per day, respectively, accounting for 33% of total emissions in the USA and approximately 80% in Australia [44, 45]. This significant variation between countries is primarily attributed to differences in their electricity mix. In France, where 67% of electricity is generated from nuclear power, the carbon intensity is notably low at 0.06 kg CO_2_​e/kWh. Conversely, Australia’s reliance on coal for electricity production results in a much higher carbon intensity of 0.56 kg CO2​e/kWh [46]. These findings underscore the necessity of conducting country-specific analyses to identify effective strategies for reducing GHG emissions. For instance, while reducing electricity consumption could have a limited impact in France due to its low-carbon electricity mix, it could be a critical lever for emission reductions in countries like Australia, where electricity generation is more carbon-intensive. Moreover, reducing the carbon intensity of electricity production in such countries — by implementing more renewable energy sources — should also be a priority [47].

In our study, waste management accounted for a minor share of emissions, contributing 4–6% depending on the patient group. We categorized waste into infectious risk waste (19%) and general waste (81%) due to their differing environmental and economic impacts. Infectious risk waste requires more complex treatment methods, resulting in pollution levels three times higher than those of general waste. Our findings suggested a higher daily waste production per patient compared to previous ICU studies. A meta-analysis of seven ICU studies reported waste generation ranging from 1.1 to 13.7 kg per patient per day [36]. Because waste recycling in ICU settings remains challenging and marginal, recent research emphasizes the need to prioritize reducing overall waste production by avoiding unnecessary use [36, 48]. Implementing such reduction strategies is essential to minimize the environmental impact of ICU operations.

Our study has several limitations that we have to acknowledge. Firstly, it was conducted in a single center with a limited number of patients. Secondly, sterilization of DM was not included in our analysis, which may lead to an underestimation of the total carbon footprint associated with DMs. Nevertheless, no specific data on sterilization was available, and a recent report from the Shift Project indicated that sterilization accounts for less than 10% of the total carbon footprint, making its contribution negligible relative to the overall CO₂ emissions per patient [49]. Thirdly, our results are not exhaustive, as we excluded patient transport, surgery, safety services, and laundry. These emission sources should be considered in future studies, given their potential impact. For example, trauma surgery has been reported to emit between 20 and 150 kg of CO₂e per procedure, which represents approximately twice the total carbon footprint observed in our mild and intermediate groups [50–52]. Nevertheless, much of our efforts were dedicated to categorizing the amount of emissions associated with the severity of patients. Fourthly, current data are incomplete, and while LCA studies are increasingly conducted in the medical field worldwide, this approach remains relatively uncommon. We therefore included a sensitivity analysis on the main emission sources to address this uncertainty. Furthermore, our decision to use a bottom-up evaluation based on our daily practice limits the generalizability of our findings, notably to other countries. However, in studies based on cost analysis, differences also depend on health care systems and countries, as well as ICU and hospital length of stays are related to the availability of beds in ICU and down-step units. Fifthly, we focused solely on carbon emissions, while acknowledging that other environmental impacts such as air, soil, and water pollution, ecotoxicity, and potential carcinogenic effects should also be considered [53].

Clinical research aims to evaluate procedures, treatments, and strategies based on well-defined outcomes. For critically ill patients, mortality remains the most assessed metric. However, other outcomes, such as quality of life, days at home, or health care associated costs, are highly relevant [54]. Our study specifically delineates the carbon cost of the first ICU day for trauma patients across varying severity levels. Based on our findings, we propose that the carbon footprint of an ICU stay be considered as a potential outcome in future critical care trials to influence the medical decision-making. Incorporating sustainability metrics into medical research could offer a more comprehensive evaluation of healthcare interventions [55, 56]. As the health care sector intensifies efforts to reduce its carbon footprint, integrating environmental impact as a measurable outcome can guide the adoption of sustainable practices without compromising the quality of care [57, 58]. In the long term, this approach could also yield significant public health benefits. Emerging data demonstrate that implementing environmental policies often results in co-benefits for population health, such as reducing respiratory and cardiovascular diseases linked to pollution [59]. Our study provides a foundation for future research comparing the global warming impacts of different management strategies. Ultimately, this could support the inclusion of the carbon footprint as a pharmaco-ecological outcome in clinical trials, helping identify optimal strategies at both individual and systemic levels.

## Conclusion

By encompassing a broad scope, we provided a holistic view of the environmental impact of critical care. Our findings highlight that while waste management is a critical focus for reducing GHG emissions, other significant contributors, such as medications and energy, should also be addressed. Transitioning to sustainable healthcare practices in ICUs requires balancing patient outcomes with economic, environmental, and social costs. Incorporating the carbon cost as a key outcome measure in ICU clinical trials, hospital certifications, and ICU management is essential to drive sustainable critical care practices.

## Supplementary Information

Below is the link to the electronic supplementary material.

Supplementary Material 1

## Data Availability

All data generated or analysed during this study are included in this published article and its Additional file.
